# Metabolic profiling of isolated mitochondria and cytoplasm reveals compartment-specific metabolic responses

**DOI:** 10.1007/s11306-018-1352-x

**Published:** 2018-03-31

**Authors:** Daqiang Pan, Caroline Lindau, Simon Lagies, Nils Wiedemann, Bernd Kammerer

**Affiliations:** 1 Center for Biological Systems Analysis, ZBSA, Albert-Ludwigs-University Freiburg, Habsburgerstraße 49, 79104 Freiburg, Germany; 2 Institute of Pharmaceutical Sciences, Albert-Ludwigs-University Freiburg, 79104 Freiburg, Germany; 3 Institute of Biochemistry and Molecular Biology, ZBMZ, Faculty of Medicine, Albert-Ludwigs-University Freiburg, Stefan-Meier-Str. 17, 79104 Freiburg, Germany; 4 Faculty of Biology, University of Freiburg, 79104 Freiburg, Germany; 5 Spemann Graduate School of Biology and Medicine (SGBM), Albert-Ludwigs-University Freiburg, 79104 Freiburg, Germany; 6 BIOSS Centre for Biological Signalling Studies, University of Freiburg, 79104 Freiburg, Germany

**Keywords:** ATP-synthase, Metabolomics, Metabolic phenotyping, Mitochondria, Succinate dehydrogenase, Yeast

## Abstract

**Introduction:**

Subcellular compartmentalization enables eukaryotic cells to carry out different reactions at the same time, resulting in different metabolite pools in the subcellular compartments. Thus, mutations affecting the mitochondrial energy metabolism could cause different metabolic alterations in mitochondria compared to the cytoplasm. Given that the metabolite pool in the cytosol is larger than that of other subcellular compartments, metabolic profiling of total cells could miss these compartment-specific metabolic alterations.

**Objectives:**

To reveal compartment-specific metabolic differences, mitochondria and the cytoplasmic fraction of baker’s yeast *Saccharomyces cerevisiae* were isolated and subjected to metabolic profiling.

**Methods:**

Mitochondria were isolated through differential centrifugation and were analyzed together with the remaining cytoplasm by gas chromatography–mass spectrometry (GC–MS) based metabolic profiling.

**Results:**

Seventy-two metabolites were identified, of which eight were found exclusively in mitochondria and sixteen exclusively in the cytoplasm. Based on the metabolic signature of mitochondria and of the cytoplasm, mutants of the succinate dehydrogenase (respiratory chain complex II) and of the F_O_F_1_-ATP-synthase (complex V) can be discriminated in both compartments by principal component analysis from wild-type and each other. These mitochondrial oxidative phosphorylation machinery mutants altered not only citric acid cycle related metabolites but also amino acids, fatty acids, purine and pyrimidine intermediates and others.

**Conclusion:**

By applying metabolomics to isolated mitochondria and the corresponding cytoplasm, compartment-specific metabolic signatures can be identified. This subcellular metabolomics analysis is a powerful tool to study the molecular mechanism of compartment-specific metabolic homeostasis in response to mutations affecting the mitochondrial metabolism.

**Electronic supplementary material:**

The online version of this article (10.1007/s11306-018-1352-x) contains supplementary material, which is available to authorized users.

## Introduction

Mitochondria have been described as the power houses of the cell due to their major function in adenosine triphosphate (ATP) synthesis, which is mainly based on the pyruvate dehydrogenase, the β-oxidation of fatty acids, the citric acid cycle (TCA cycle), and the respiratory chain (RC) (Nunnari and Suomalainen 2012; van der Bliek et al. 2017). Defects in mitochondria are linked to human diseases such as mitochondrial encephalomyopathy, Alzheimer’s disease, diabetes and cancer (Schapira 2012; Gaude and Frezza 2014; Paglia et al. 2016). Therefore, it is crucial to identify mechanisms of mitochondrial diseases so that possible therapies can be developed.

Mass spectrometry (MS) based metabolomics has become a promising research tool for various biological systems, including bacteria, plants, mammalian cells, tissues and body fluids (Fiehn et al. 2000; Liesenfeld et al. 2013; Sana et al. 2013; Willmann et al. 2016). Genetic and other pathological or environmental changes can result in alterations of metabolites, which can be revealed by metabolomics. Alterations of metabolites have been reported in blood plasma, urine of cancer patients and cancer cell lines, providing promising possibilities of biomarker discoveries (Bullinger et al. 2007; Frickenschmidt et al. 2008; Asiago et al. 2010; Shen et al. 2013; Armitage and Barbas 2014; Willmann et al. 2015). Additionally, metabolomics helps in understanding cellular mechanisms by showing the metabolic status of drug-treated or non-treated cancer cells (Jain et al. 2012; Budczies et al. 2015; Pan et al. 2016). Since metabolomics has been described as the link between genotype and phenotype (Fiehn 2002), Stefely et al. (2016) have profiled 174 yeast strains with different single gene knockouts to elucidate the mitochondrial protein functions. However, whole cells were applied in the profiling, which could not uncover the compartment-specific metabolic signature. In contrast, van Vranken and Rutter (2016) valued subcellular compartment metabolic profiling by coining the term ‘the whole (cell) is less than the sum of its parts’.

Compartment-specific mitochondrial metabolite levels were already analyzed by Siess et al. (1978) for TCA cycle related metabolites and by Ross-Inta et al. (2008) for mitochondrial amino acids. Although isolation of mitochondria has been established and optimized in several research groups (Meisinger et al. 2006; Boldogh and Pon 2007; Fernández-Vizarra et al. 2010), mitochondrial metabolomics using modern MS equipment has not been applied widely due to technical difficulties. One of the first studies analyzing isolated mitochondria by state of the art metabolomics methods was published by Roede et al. (2012). They could distinguish male from female and wild type from mutant. This was the first time that phenotypes could be discriminated from each other based on the mitochondrial metabolic signature. Although only part of the detected features could be annotated as metabolites based on m/z and retention time (RT), the remaining features indicated a large amount of unidentified known and/or unknown metabolites in mitochondria. A compartment-specific metabolomics study was conducted by filtration of Chinese hamster ovary cells and revealed a very low ATP pool within mitochondria (Matuszczyk et al. 2015). Recently, Chen et al. (2016) determined absolute concentrations for over 100 mitochondrial matrix metabolites in comparison to whole cells employing immunopurification of mitochondria using an artificial epitope tag fused to an outer membrane anchor. In this study a higher concentration was observed for most of the metabolites in whole cells compared to mitochondria. Besides that, the metabolic responses to RC inhibitors were in mitochondria stronger compared to whole cells. Hence, it is reasonable to expect that metabolic alterations are compartment-specific. By means of our approach compartment-specific metabolic signatures could be detected, which can be related to specific metabolic alterations in mitochondrial mutants.

## Materials and methods

### Yeast

*Saccharomyces cerevisiae* strains *atp4*Δ (4333), *sdh2*Δ (1996) and the corresponding wildtype (WT) BY4741 (1354) with the genotype MATa, ura3Δ0, leu2Δ0, his3Δ1, met15Δ0 (Baker Brachmann et al. 1998) were obtained from Euroscarf. Yeast cells were cultured in liquid synthetic galactose media (0.67% [w/v] yeast nitrogen base without amino acids (Becton, Dickinson and Company, MD, USA), 0.77 g/L SC amino acids (MP Biomedicals, USA), 2% [w/v] galactose (Sigma-Aldrich)) at 30 °C under shaking at 130 rpm. Cells were harvested in exponential phase.

### Isolation of mitochondria and the cytoplasm

Based on the protocol from Meisinger et al. (2006) and the isolation buffer used by Corcelli et al. (2010), mitochondria were isolated with optimized MS compatible KClEM isolation buffer [180 mM KCl, 1 mM EDTA and 5 mM MOPS-KOH (pH 7.2)]. Briefly, after incubation in dithiothreitol buffer, yeast cells were washed with KClEM buffer and treated with zymolyase (Seigaku), subsequently washed and pottered in ice-cold KClEM buffer. The nuclei were pelleted by subsequently increasing the centrifugal force 250×*g* for 2 min, 1000×*g* for 4 min and 1900×*g* for 4 min in a single centrifugation run. The post nuclear supernatant was subjected to a 15 min 16,800×*g* centrifugation step to pellet the mitochondrial fraction. The post mitochondrial supernatant was collected and regarded as remaining cellular cytoplasm for further analysis. The protein concentration of isolated mitochondria was determined using the Bradford assay (Roth). Mitochondria were aliquoted into 1 mg aliquots and the corresponding fraction of cytoplasm was calculated based on the quantity of mitochondria and the volume of the cytoplasmic fraction. To test the integrity of the isolated mitochondria, 30 µg mitochondria were thawed on ice and treated with an indicated amount of Proteinase K (Roche) for 10 min on ice. Proteinase K was inactivated by addition of 2.4 mM PMSF (Phenylmethylsulfonylfluorid, Roth) and further incubated on ice for 10 min. Mitochondria were pelleted for 10 min at 20,817×*g* (14,000 rpm, 5417R, Eppendorf) at 4 °C and washed with 200 µL isolation buffer containing 1 mM PMSF.

### SDS-PAGE and western blotting

For SDS-PAGE analysis, mitochondria were resuspended in Laemmli buffer (60 mM Tris (MP Biomedicals), pH 6.8, 2% [w/v] SDS (SDS, Serva), 10% [v/v] glycerol (Honeywell Research Chemicals), 0.02% [w/v] bromphenol blue (Sigma)) containing 1–2 mM PMSF and 1% [v/v] 2-mercaptoethanol and incubated for 15 min at 60 °C with shaking at 1400 rpm. Samples were analyzed by using 10% Tris–Tricine polyacrylamide gels containing 10% polyacrylamide (49.5% [w/v] acrylamide (Roth), 3% [w/v] bisacrylamide (Serva)). Gel electrophoresis was performed in anode (0.2 M Tris/HCl, pH 8.9) and cathode (0.1 M Tris, 0.1 M Tricine (Roth), 0.1% [w/v] dodecylsulfate-Na-salt (SDS, Serva), pH 8.25) buffer at 70 mA, 600 V for 3.5 h. Afterwards, proteins were transferred to PVDF membranes (Immobilon-P, Millipore) by performing a standard semi-dry western blot transfer at 250 mA, 25 V for 2.5 h in western transfer buffer (20 mM Tris, 150 mM glycine (MP Biomedicals), 0.02% [w/v] SDS, 20% [v/v] methanol). Membranes were stained in 0.2% Coomassie R250 (Roth), 10% [v/v] acetic acid (Roth) and 30% [v/v] ethanol and subsequently destained in 100% methanol and washed shortly in TBST [200 mM Tris/HCl, pH 7.5, 1.25 M CaCl_2_, 0.1% Tween20 (Sigma)] before they were incubated for 1 h in 5% [w/v] fat-free dried milk powder (Frema Reform) in TBST at room temperature. Membranes were shortly washed in TBST and then incubated for 3 h in the indicated primary antibodies at room temperature. After three 5 min washing steps in TBST, the membranes were incubated for 1 h at room temperature in secondary anti-rabbit IgG antibody (Sigma), diluted 1:5000 in 5% [w/v] fat-free dried milk powder in TBST, which is coupled to a horseradish peroxidase. After three 5 min washing steps in TBST, membranes were incubated in ECL solution (Haan and Behrmann 2007) and chemiluminescence signals were detected by a LAS-4000 camera system (Fujifilm). Antibodies used in this study are listed by antigen, dilution and number: Tom70, 1:500 TBST + 5% milk, GR657-4; Tom40, 1:500 TBST + 5% milk, GR168-4; Tim44, 1:400 TBST + 5% milk, GR127-6; Pam17, 1:500 TBST + 5% milk, GR3885-5.

### Metabolite extraction, GC–MS analysis and data evaluation

Mitochondrial pellets corresponding to 1 mg protein and half of the volume of each corresponding cytoplasm were applied to metabolic profiling in technical triplicate. The results were reproduced by analyzing different biological preparations. The cytoplasm was dried in a Concentrator plus vacuum rotator (Eppendorf, Germany) before 1 mL extraction medium (− 20 °C methanol/water (v/v: 90/10) containing 1 µg/mL of β-phenylglucose and ribitol as internal standards) was added to each mitochondrial or cytoplasmic pellet. Samples were incubated at 4 °C and 1200 rpm for 10 min after short vortex. After centrifugation at 21,000×*g* and 4 °C for 10 min, the metabolite-containing supernatants were transferred to new reaction tubes and dried in a Concentrator plus vacuum rotator and stored under nitrogen atmosphere at − 80 °C until derivatization.

Based on the protocol of Fiehn (2006), sample derivatization, gas chromatography–mass spectrometry (GC–MS) analysis and data evaluation were performed as described by Pan et al. (2016). Briefly, the prewarmed pellets were derivatized by shaking with 20 µL 20 mg/mL methoxyamine hydrochloride (Sigma-Aldrich, Germany) in pyridine (Sigma-Aldrich, Germany) at 1200 rpm and 28 °C for 90 min and, after short centrifugation, with 50 µL *N*-methyl-*N*-trimethylsilyltrifluoroacetamide (Sigma-Aldrich, Germany) at 1200 rpm and 37 °C for 30 min.

An HP-5MS capillary column (Agilent, Germany) with the dimension of 60 m × 0.25 mm × 0.25 µm was used for GC separation with helium as carrier gas. The oven temperature was held at 80 °C for the first 2 min, then increased to 320 °C within 50 min and kept at 320 °C for 10 min. A C10–C40 *n*-alkane standard mixture (Neochema, Bodenheim, Germany) was applied at the beginning for retention index calculation. All GC–MS data files were processed with AMDIS (Version 2.71, National Institute of Standards and Technology, Gaithersburg, MD, USA) and the web-based tool SpectConnect (Styczynski et al. 2007) to perform the peak picking, spectral deconvolution and compound identification. Annotation of metabolites was performed according to the retention index deviation (< 5%) and spectra match score (> 750) in NIST (Version 2.2, National Institute of Standards and Technology, Gaithersburg, MD, USA), GolmDB (Kopka et al. 2005) and Fiehnlib (Kind et al. 2009) databases. Annotated metabolites were normalized by internal standard and protein amount. Finally, MetaboAnalyst 3.0 (Xia et al. 2015) was used for statistical analysis.

## Results and discussion

### Inner membrane integrity of isolated mitochondria in mass spectrometry compatible potassium chloride buffer

**Fig. 1:**
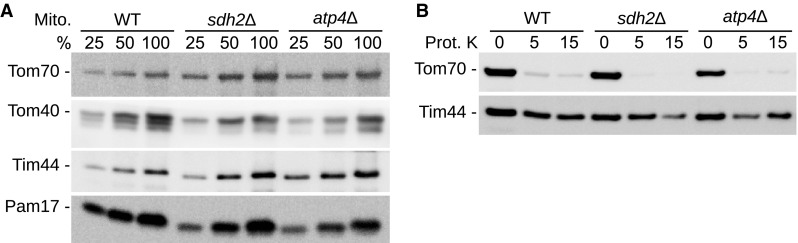
Integrity of the mitochondrial inner membrane. **a** Mitochondrial protein steady state levels were analyzed by SDS-PAGE and western blotting with antibodies directed against the indicated proteins. **b** Isolated mitochondria suspended in potassium chloride based isolation buffer were treated with Proteinase K (Prot. K) and analyzed as described in panel (**a**)

Since *S. cerevisiae* has been used as a system for modeling mitochondrial diseases (Goffeau et al. 1996; Herrgard et al. 2008; Lasserre et al. 2015), baker’s yeast was selected to analyze the compartment-specific metabolic alteration of mutants affecting the mitochondrial metabolism. In addition to isolated mitochondria of wild type (WT) and RC mutants, the corresponding cytoplasm was also analyzed by GC–MS based metabolic profiling. Yeast cells were cultivated in galactose containing medium, inducing a respiratory growth behavior in the strain BY4741 (Morgenstern et al. 2017). Chen et al. (2016) has described the incompatibility of traditional mitochondrial isolation buffers with liquid chromatography–MS and applied a potassium chloride based isolation buffer. Along with their findings, sugars as osmolytes used in the traditional mitochondrial isolation buffers were also found in our approach to be incompatible with GC–MS. We established a mitochondria isolation procedure by differential centrifugation using the potassium chloride buffer established by Corcelli et al. (2010) to achieve a direct comparison of mitochondrial and cytoplasmic metabolites. To avoid unnecessary equilibration of metabolites across the mitochondrial membrane channels and transporters, we minimized the isolation time after disruption of the cells to only two centrifugation steps: first to pellet the nuclei by centrifugation for a total of 10 min and second to pellet mitochondria for 15 min. The post-mitochondrial supernatant was collected as corresponding cytoplasm consisting of the cytosol and the other remaining organelles. The mitochondrial and cytoplasmic fractions were then used to detect compartment-specific metabolic signatures. To prove the feasibility of our approach we selected two classical mitochondrial mutants: The electron transport chain and TCA cycle mutant strain with a deletion of the succinate dehydrogenase (complex II) subunit 2 (*sdh2*Δ) and the F_O_F_1_-ATP synthase (complex V) subunit 4 (*atp4*Δ) deleted strain. Mitochondrial proteins were subjected to SDS-PAGE analysis and subsequent western blotting, indicating similar levels of translocase of the outer mitochondrial membrane (TOM) proteins Tom70 and Tom40 and translocase of the inner mitochondrial membrane (TIM) protein Tim44 and of the presequence translocase associated import motor (PAM) protein Pam17 (Wiedemann and Pfanner 2017) (Fig. 1a). To test the integrity of the mitochondrial inner membrane upon isolation in MS compatible potassium chloride buffer, isolated mitochondria were treated with increasing concentrations of Proteinase K (Prot. K). The protease was inactivated by phenylmethylsulfonyl fluoride before mitochondrial proteins were analyzed by SDS-PAGE and western blotting. The outer membrane protein Tom70, which contains a large cytosolic domain exposed to the outside of mitochondria, is efficiently degraded by addition of the external protease. In contrast, the mitochondrial matrix protein Tim44 remains largely unaffected by protease treatment, indicating that the mitochondrial inner membrane remains intact (Fig. 1b).

### Compartment-specific metabolic signature in mitochondria and cytoplasm

**Fig. 2:**
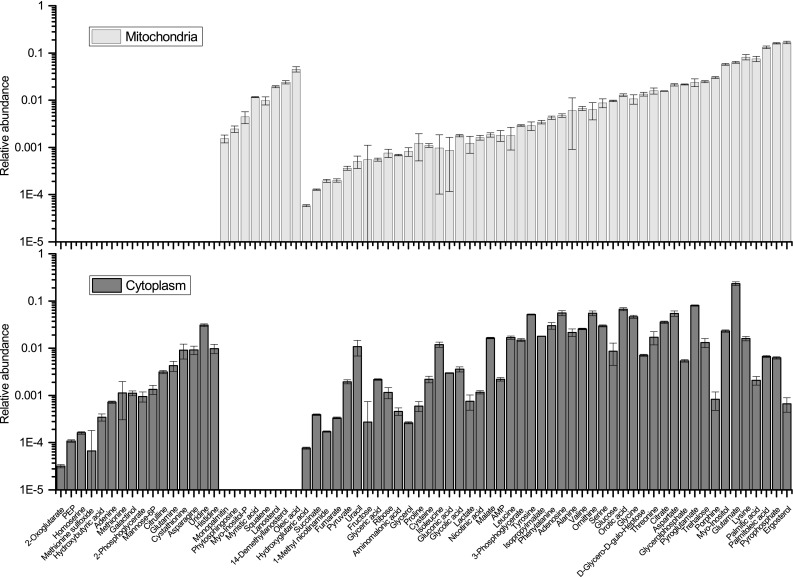
Bar charts indicating the relative abundance of identified metabolites in mitochondria and the cytoplasm of wild type (mean ± SD, n = 3). Eight metabolites were found exclusively in mitochondria and sixteen exclusively in the corresponding cytoplasm. *PEP* phosphoenolpyruvate, *Mannose-6P* mannose-6-phophate, *Myo-Inositol-P* myo-inositol-phosphate, *AMP* adenosine monophosphate

Isolated mitochondria and the corresponding cytoplasm of WT were analyzed concomitantly by GC–MS based metabolic profiling. Seventy-two metabolites were identified altogether, of which eight were found exclusively in mitochondria while sixteen were found exclusively in the cytoplasm, indicating compartment-specific distribution of metabolites in our preparations (Fig. 2). These findings are in good agreement with the mitochondrial isolation procedure by immunopurification (Chen et al. 2016). This indicates that within the time of the mitochondrial isolation, compartment-specific metabolite distribution was preserved. The mitochondria specific metabolites are mainly fatty acids and steroid intermediates. Given that many metabolites show higher levels in the cytoplasmic fraction compared to mitochondria (Chen et al. 2016), it is possible that metabolic alterations of mutants, affecting the mitochondrial metabolism, may be buried in the metabolite pool of total cells.

### Mutations induce compartment-specific metabolic regulation

**Fig. 3:**
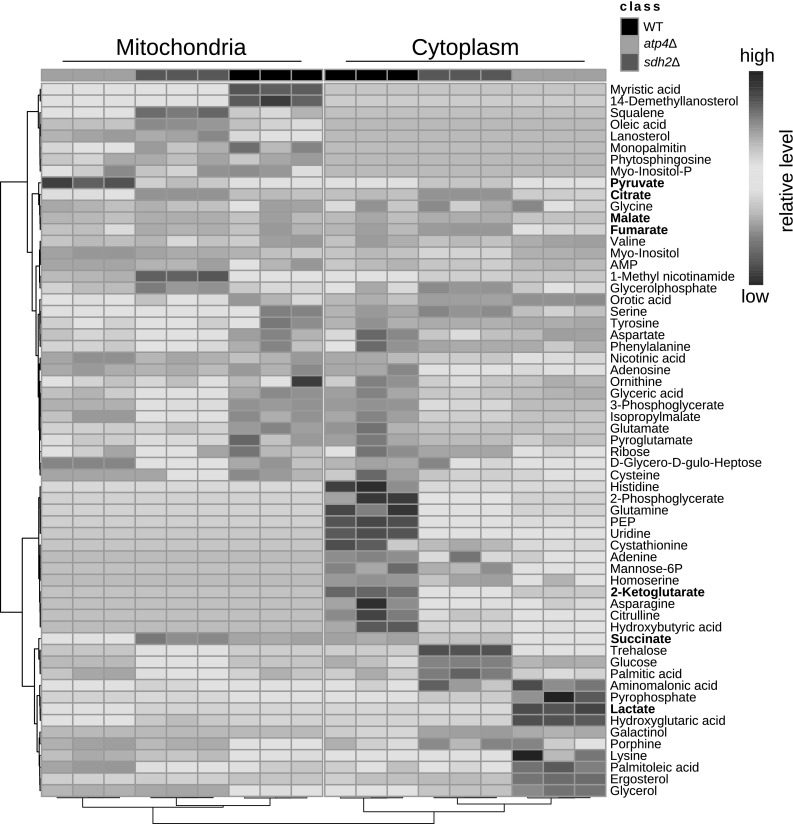
Heatmap shows the fold changes of metabolites in mitochondria and the cytoplasm of WT and the two mutants, *atp4*Δ and *sdh2*Δ. Lactate, pyruvate and detected TCA cycle intermediates are highlighted in bold text. Only 61 out of 72 identified metabolites are shown here with a q value < 0.001

To analyze if compartment-specific metabolic responses, caused by changes of the mitochondrial metabolism, can be detected, mitochondria and the corresponding cytoplasmic fractions of WT, *atp4*Δ and *sdh2*Δ yeast cells were subjected to GC–MS. Citrate synthase is used to normalize mitochondrial data in many studies (Mogensen et al. 2007; Chen et al. 2016). However, Morgenstern et al. (2017) showed that the level of citrate synthase 1 (Cit1) is variable depending on the growth conditions. The normalization using citrate synthase would be problematic given that *ATP4* and *SDH2* are functionally connected to the TCA cycle and their deletions will impact the growth conditions, the level of mitochondrial citrate synthase (Cit1) and its activity. Therefore, the GC–MS data was normalized, in addition to internal standard, by the protein content of the mitochondrial fraction, whose amount was verified by employing specific antibodies against four selected mitochondrial marker proteins Tom70, Tom40, Tim44 and Pam17. The fold change (FC) of metabolites in mutants compared to WT is regarded as the metabolic response to the mutation of a specific gene. In order to find significantly altered metabolites, analysis of variance (ANOVA) was performed, followed by false discovery rate (FDR) to exclude the false significant metabolites. Sixty-one metabolites showed significantly (p < 0.001, q < 0.001) altered FCs (see Supplementary Table S1). Based on these FCs, a heatmap (Fig. 3) was generated, presenting the change of each metabolite in mitochondria and the cytoplasm of *atp4*Δ and *sdh2*Δ compared to WT. Consequently, the alteration of metabolites in *atp4*Δ and *sdh2*Δ are opposing for a large fraction of the detected metabolites between mitochondria and the cytoplasm (Fig. S1), indicating different metabolic activities in both compartments. Moreover, both mutants show a specific metabolite alteration pattern. As a result of these mutations affecting mitochondrial respiration, not only the TCA cycle, but also amino acid metabolism, fatty acid metabolism, glycolysis and nucleotide metabolism were affected.

**Fig. 4:**
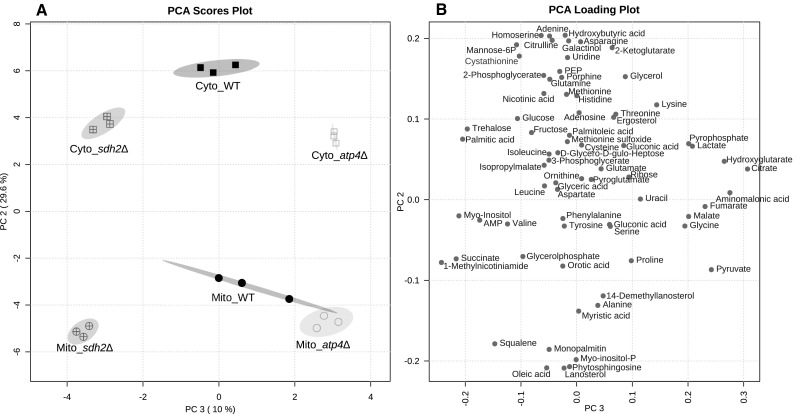
**a** Principal component (PC) analysis plot and **b** its loading map. PC2 discriminates mitochondria (Mito) from the cytoplasm (Cyto), while PC3 discriminates *atp4*∆ and *sdh2*∆ from each other and WT. The loading map displays how the individual metabolites contribute to the discrimination. The bigger the absolute PC value of a metabolite is, the more it contributes to the discrimination in this PC

Based on the detected metabolites principal component analysis (PCA) can discriminate both the mitochondrial and the cytoplasmic compartments from each other. As shown in Fig. 4a, PC2 discriminates mitochondria from the cytoplasm. Based on the metabolic alterations caused by the deletion of *ATP4* or *SDH2*, PC3 discriminates *atp4*∆ and *sdh2*∆ from each other and from WT. The loading map with the values for PC2 and PC3 in Fig. 4b shows how single metabolites influenced the discrimination of compartments and strains. Consequently, the compartment-specific metabolites (negative PC2 value for mitochondria-specific and positive for cytoplasm-specific) as observed in Fig. 2 contributed to the discrimination in PC2, while TCA cycle related intermediates like pyruvate, citrate, fumarate and malate made a major contribution to PC3, highlighting their dysregulation in the mutant mitochondria. Besides that, they are also clustered together in the heatmap, displaying a similar regulation pattern.

**Fig. 5:**
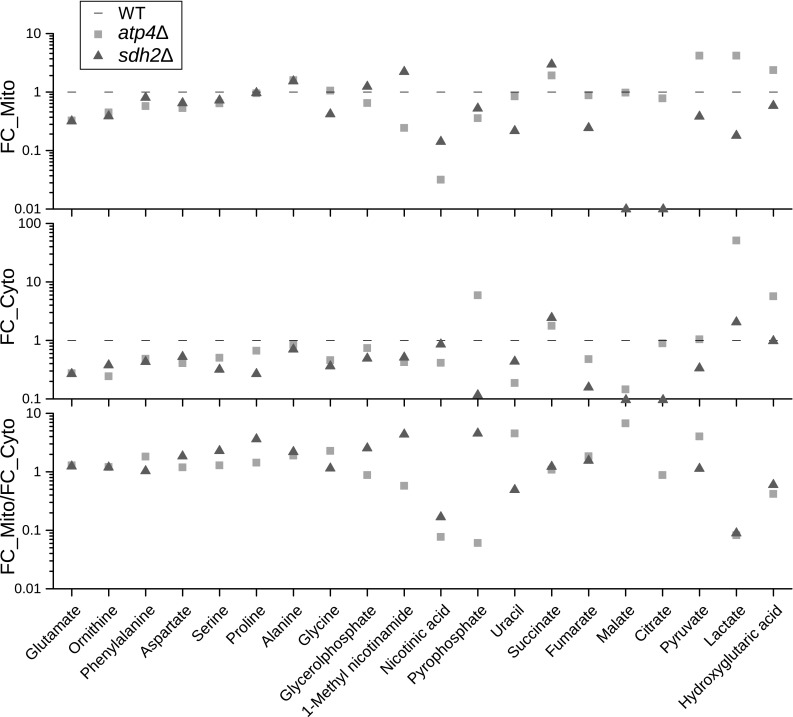
Overview of fold changes (FC) of altered amino acids, TCA cycle intermediates and several other metabolites in mitochondria and cytoplasm of *atp4*∆ and *sdh2*∆ cells. Not-detected metabolites are shown on the horizontal axes. FC_Mito/FC_Cyto indicates the compartment-specific metabolic alterations. The bigger the deviation of the value from 1 is, the more significant the difference is

Using this compartment-specific metabolomics approach we identified a number of metabolites, which show a differential regulation pattern between mitochondria and the cytoplasm (Fig. 5). The levels of amino acids like glutamate, ornithine, phenylalanine, aspartate and serine are reduced in both compartments of *atp4*∆ and *sdh2*∆. However, proline showed significantly reduced levels in the cytoplasm and relatively normal mitochondrial levels compared to WT. In contrast to the generally reduced amino acids, the levels of alanine are slightly decreased in the cytoplasm of *atp4*∆ and *sdh2*∆ and concomitantly increased in mitochondria of both mutants, whereas glycine is specifically reduced in *sdh2*∆ and normal for *atp4*∆ mitochondria.

The gradient of nicotinic acid was dramatically altered in the mutants, with five to ten times reduced levels within mitochondria. The levels of the primary nicotinamide metabolite 1-methyl-nicotinamide were reduced in the cytoplasm of both analyzed mutants. In *atp4*∆ mitochondria levels are ~ 4 times lower compared to WT. In contrast, in *sdh2*∆ mitochondria levels of 1-methyl-nicotinamide are increased by more than two-fold compared to WT, leading to opposing gradients between mitochondria and the cytoplasm. For the nucleic acid base uracil the levels were reduced by four times in the cytoplasm in *atp4*∆ compared to WT. In contrast, for *sdh2*∆ the uracil ratio was shifted to the opposite direction with lower levels in mitochondria. Another very spectacular example of compartment-specific differential levels is pyrophosphate with reduced mitochondrial levels in both mutants, while in *sdh2*∆ cytoplasmic pyrophosphate was even more reduced compared to ~ 6 times increased cytoplasmic levels in *atp4*∆ leading again to opposing gradients in both mutants. Interestingly, the alterations of TCA cycle intermediates were not only compartment-specific but also strain-specific. This will be discussed in the following paragraph.

### The TCA cycle is interrupted in *sdh2*∆ but maintained in *atp4*∆ cells

Deletion of the succinate dehydrogenase gene *SDH2* and the FoF_1_-ATP-synthase gene *ATP4* causes a loss of function in respiratory complex II and complex V, resulting in impaired mitochondrial respiration. Consequently, TCA intermediates and related metabolites were affected not only in mitochondria but also in the cytoplasm. As shown in Fig. 5, succinate is the single up-regulated metabolite with relation to the TCA cycle in both mutants and compartments. As succinate is oxidized to fumarate by succinate dehydrogenase, deletion of *SDH2* resulted in an accumulation of succinate and a concomitant decrease of fumarate and malate in mitochondria. Although the F_O_F_1_-ATP-synthase does not have a direct connection to succinate, its mutation also caused a significant accumulation of succinate. Since deletion of *ATP4* interrupts ATP synthesis accompanied by accumulation of protons in the intermembrane space, elevated level of reduced coenzyme Q results in complex II inhibition and accumulation of succinate. As reviewed by Tretter et al. (2016), succinate is not only an intermediate in metabolism, but also plays an important role in signal transduction, ROS, hypoxia and tumorigenesis, which can induce massive metabolite alterations besides TCA cycle intermediates. Except for succinate, TCA cycle intermediates showed completely different regulation patterns between the two mutants. Fumarate, malate and citrate were down-regulated in both compartments of *sdh2*∆, while normal levels were detected in mitochondria of *atp4*Δ. Since deletion of *SDH2* interrupted the TCA cycle, its intermediates decreased overall, especially citrate and malate, which could not be detected either in mitochondria or in cytoplasm. Similar results were shown by Cardaci et al. (2015), demonstrating that SDH-deficient cells use pyruvate carboxylation to synthesize aspartate.

## Conclusion

**Fig. 6:**
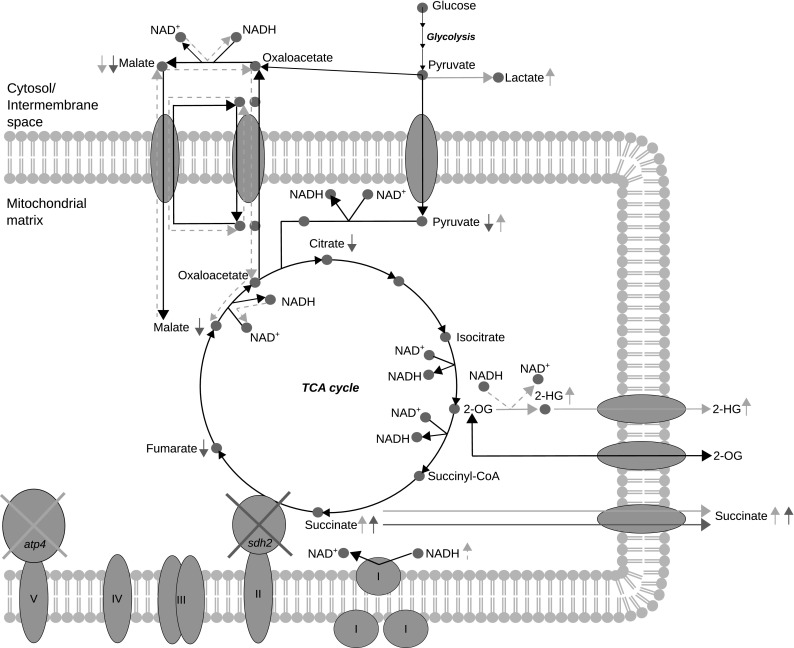
Overview of metabolic alterations in mitochondria of *sdh2*Δ and *atp*4Δ mutants. The relevant metabolic pathways are shown by solid black lines. The metabolic changes caused by the deletion of *SDH2* or *ATP4* are displayed by either purple for *sdh2*Δ or deep yellow for *atp4*Δ. The colored solid lines indicate the observed metabolic dysregulations and the dashed lines indicate the putative related metabolic changes (hypothesis of the authors)

Since the metabolic response of mutations affecting the mitochondrial matrix metabolism can be buried in the pool of cytosolic metabolites, we established a compartment-specific metabolic approach analyzing isolated mitochondria and their corresponding cytoplasmic fraction by applying GC–MS based metabolomics. Deletion of genes encoding the mitochondrial electron transport chain subunit Sdh2 and the FoF_1_-ATPase subunit Atp4 resulted in massive metabolic alterations in both mitochondria and cytoplasm (Figs. 3, 5). The decrease of malate in the cytosol of *atp4*Δ may be the result of accumulated mitochondrial NADH, triggering the cells to export reducing equivalents via reverse malate shuttle (Fig. 6). GOT1, as part of the malate-aspartate shuttle, has been described by Birsoy et al. (2015) to generate aspartate from oxaloacetate in the cytosol upon electron transport chain inhibition (complex I and III). Together with our results, it is tempting to speculate that cells can reverse this shuttle not only to produce aspartate, but also to export NADH. The exported malate is oxidized to oxaloacetate, generating cytosolic NADH, which can contribute to the reduction of pyruvate to lactate (highly accumulated in cytoplasm, see Fig. 5), indicating a metabolic state similar to anaerobic growth. In contrast to *atp4*Δ, the levels of cytoplasmic lactate are not significantly increased and mitochondrial lactate is even strongly reduced in *sdh2*Δ cells. Even though complex II activity is blocked in *sdh2*Δ, reducing equivalents can be transferred to the electron transport chain via the NADH-dehydrogenases in the inner membrane (Marres et al. 1991; Luttik et al. 1998). This is supported by Kwon et al. (2015), showing a slightly decreased oxygen consumption rate in *sdh2*Δ cells and no oxygen consumption in *atp4*Δ cells.

Besides lactate, 2-hydroxyglutarate was specifically elevated in the cytoplasm of *atp4*Δ cells. Recently, the metabolic pathway involving 2-hydroxyglutarate for shuttling reducing equivalents from cytosol to mitochondria was described in yeast (Becker-Kettern et al. 2016). Considering the accumulation of reducing equivalents in *atp4*Δ mitochondria, this import pathway may be employed reversely to export reducing equivalents from mitochondria to the cytosol. Reduction of mitochondrial 2-oxoglutarate generates 2-hydroxyglutarate, followed by transport to cytosol. Oxidation to 2-oxoglutarate by the mitochondrial retrograde response induced transhydrogenase Dld3 (Chelstowska et al. 1999), which concomitantly reduces pyruvate to lactate (Becker-Kettern et al. 2016), could also contribute to the high levels of cytosolic lactate in *atp4*Δ cells. This function is in agreement with the proposed function for Dld3 to reduce the generation of NADH in respiratory deficient cells (Liu and Butow 2006). A model was generated in Fig. 6, describing the observed metabolic response in mitochondria of *atp4*∆ and *sdh2*∆ cells. Taken together, this study shows that a compartment-specific metabolomics analysis is superior for the analysis and interpretation of pathological metabolic alterations and will be an important asset for future metabolic research.

## Electronic supplementary material

Below is the link to the electronic supplementary material.

**Figure S1** Comparison of metabolic responses of mitochondria and cytoplasm in *atp4*Δ and *sdh2*Δ. Data is adapted from figure 3 and only in both compartments detected metabolites are shown. (EPS 508 KB)

**Supplementary Table S1** Including raw data and all the processed data. (XLSX 577 KB)
